# Different α-synuclein prion strains cause dementia with Lewy bodies and multiple system atrophy

**DOI:** 10.1073/pnas.2113489119

**Published:** 2022-02-03

**Authors:** Jacob I. Ayers, Joanne Lee, Octovia Monteiro, Amanda L. Woerman, Ann A. Lazar, Carlo Condello, Nick A. Paras, Stanley B. Prusiner

**Affiliations:** ^a^Institute for Neurodegenerative Diseases, Weill Institute for Neurosciences, University of California, San Francisco, CA 94158;; ^b^Department of Neurology, Weill Institute for Neurosciences, University of California, San Francisco, CA 94158;; ^c^Division of Biostatistics, University of California, San Francisco, CA 94158;; ^d^Division of Oral Epidemiology and Dental Public Health, University of California, San Francisco, CA 94143;; ^e^Department of Biochemistry and Biophysics, University of California, San Francisco, CA 94158

**Keywords:** neurodegeneration, dementia with Lewy bodies, synucleinopathies, strains, prions

## Abstract

The α-synuclein protein can adopt several different conformations that cause neurodegeneration. Different α-synuclein conformers cause at least three distinct α-synucleinopathies: multiple system atrophy (MSA), dementia with Lewy bodies (DLB), and Parkinson’s disease (PD). In earlier studies, we transmitted MSA to transgenic (Tg) mice and cultured HEK cells both expressing mutant α-synuclein (A53T) but not to cells expressing α-synuclein (E46K). Now, we report that DLB is caused by a strain of α-synuclein prions that is distinct from MSA. Using cultured HEK cells expressing mutant α-synuclein (E46K), we found that DLB prions could be transmitted to these HEK cells. Our results argue that a third strain of α-synuclein prions likely causes PD, but further studies are needed to identify cells and/or Tg mice that express a mutant α-synuclein protein that is permissive for PD prion replication. Our findings suggest that other α-synuclein mutants should give further insights into α-synuclein prion replication, strain formation, and disease pathogenesis, all of which are likely required to discover effective drugs for the treatment of PD as well as the other α-synucleinopathies.

## Significance

Dementia with Lewy bodies (DLB) and multiple system atrophy (MSA) are caused by α-synuclein prions that differ from each other and from those causing Parkinson’s disease (PD). DLB prions differ in their infectivity from those causing MSA or PD. The wild-type, normal version of the α-synuclein protein has the acidic amino acid glutamate (E) at residue 46, while in cases of inherited PD, it is mutated to the basic amino acid lysine (K). Using genetically engineered α-synuclein, we identified unique conditions for propagating MSA and DLB prions. Being able to distinguish among strains of naturally occurring α-synuclein prions may make it possible to develop strain-specific therapeutics for MSA, DLB, and PD.

More than 200 y ago, James Parkinson described the disease that bears his name (1). Little progress was made in deciphering the cause of Parkinson’s disease (PD) for nearly a century before Fritz Lewy discovered inclusions in the brains of PD patients (2). In his initial manuscript, he described these inclusions as eosinophilic and insoluble in alcohol, chloroform, and benzene, consistent with the presence of a major protein component. Konstantin Tretiakoff described the abundance of these inclusions in the substantia nigra in PD 2 y later and named them Lewy bodies (3).

The putative proteins of Lewy bodies remained undefined for almost a century until genetic linkage studies elucidated the relationship between types of familial PD (fPD) and mutations in the gene encoding the α-synuclein protein, *SNCA* (4). Soon thereafter in 1997, α-synuclein antibodies were found to bind Lewy bodies, the pathological hallmark of PD and dementia with Lewy bodies (DLB) (5). The following year, multiple groups reported α-synuclein immunostaining of glial cytoplasmic inclusions (GCIs) found in the brains of deceased patients with multiple system atrophy (MSA) (6–8).

The increasing phenotypic similarities among PD, DLB, MSA, and some other transmissible neurodegenerative diseases (NDs) have led many investigators to classify the synucleinopathies as prion diseases, despite some skeptics. Arguing against the possibility that PD might be a prion disorder was an early study reporting the absence of disease transmission following intracerebral inoculation of PD brain extracts to apes and monkeys (9). However, similar to other studies of prion proteins such as PrP, amyloid-β, and tau, studies investigating α-synuclein have focused on the possibility that as the structure of α-synuclein misfolds and adopts a β-sheet–rich structure, it acquires toxic properties that lead to the synucleinopathies. A few studies have investigated the mechanism by which α-synuclein acquires a β-sheet–rich structure characteristic of prions (10–14).

A decisive step toward elucidating the cause of the synucleinopathies occurred when brain homogenates from deceased MSA patients were found to transmit disease to transgenic (Tg) (SNCA*A53T) M83^+/−^ mice, hereafter referred to as TgM83^+/−^ mice (15, 16). All the TgM83^+/−^ mice inoculated with human MSA brain homogenates developed a progressive neurological disorder manifested as diminished motor movements followed by paralysis. On postmortem examination, the brains of these inoculated TgM83^+/−^ mice were found to contain α-synuclein*A53T aggregates within the CNS. The median incubation period for the MSA-inoculated TgM83^+/−^ mice was ∼120 d postinoculation. Remarkably, brain samples from deceased MSA patients that had been stored in formalin for as long as 20 y were still found to be infectious when passaged in this Tg mouse line (17).

Complementary to the TgM83^+/−^ mouse model, a cultured HEK293T cell line was developed that overexpresses a yellow fluorescent protein (YFP)–tagged human α-synuclein protein containing the A53T mutation (α-syn140*A53T-YFP) (16). With this model, it became possible to measure the activity of α-synuclein prions in samples from MSA brains after only 3 d of incubation by quantifying the formation of fluorescent α-synuclein aggregates that manifested as intracellular puncta (16, 18, 19). In a subsequent study, it was discovered that HEK cells expressing the α-synuclein E46K mutation prevented replication of MSA prions, while cells expressing either the wild-type (WT) protein or the A30P mutation were capable of supporting MSA prion formation (18). The transmission of brain homogenates from PD cases was also examined but found to neither transmit disease to TgM83^+/−^ mice nor infect the α-syn140*A53T-YFP cell line (16).

Although several laboratories have reported successful transmission of PD prions using cultured HEK cells expressing α-syn140*A53T, we have been unable to confirm these findings (20, 21). Protein misfolding cyclic amplification (PMCA) and other in vitro approaches have been used to differentiate MSA α-synuclein prions from those causing PD and DLB (11, 12, 22, 23). Together, these findings have led us, and other investigators, to hypothesize that alternative conformations of α-synuclein prions cause PD, DLB, and MSA (11, 15, 16, 21, 22, 24–26).

To investigate the potential strain-specific differences among MSA, DLB, and PD α-synuclein prions, we tested their ability to replicate in our previously described cultured cell models (18). By utilizing a modified protocol to purify α-synuclein prions from patient brain samples, we detected DLB α-synuclein prion activity. The low α-synuclein prion levels in PD samples prevented reliable characterization. Using α-synuclein HEK cell lines, we then asked whether PD or DLB prion formation was similar to that found in MSA. Contrary to the finding that MSA was unable to infect the α-syn140*E46K cell line, DLB α-synuclein prions were capable of replicating in all four of the α-synuclein cell models tested. While the infectivity from most of the DLB samples was sufficient to perform studies using our α-synuclein-YFP–overexpressing HEK cells, the levels of PD prions remained insufficient for most studies. Our data argue that α-synuclein prions that accumulate and lead to MSA are conformationally distinct than those causing DLB. Deciphering the molecular mechanism in which single–amino-acid substitutions exquisitely control α-synuclein prion strain replication will likely be pivotal in reliably establishing the epidemiology of different synucleinopathies. This mechanism would also advance the discovery of drugs that effectively slow neurodegeneration caused by MSA, DLB, and possibly PD prions.

## Results

### Enhanced Precipitation of MSA Prions.

In earlier studies, we reported that α-synuclein prions isolated from the brains of deceased MSA patients could infect HEK293T cells expressing α-syn140*A53T-YFP, resulting in the formation of bright fluorescent intracellular puncta (16, 19). Though crude MSA brain homogenates could induce a modest level of prion infectivity in cultured HEK cells, phosphotungstic acid (PTA) precipitation significantly enhanced synuclein prion infectivity (16, 19). When brain homogenates from PD patients were tested, neither crude brain homogenates nor PTA-precipitated fractions were found to be infectious (16, 19). In an attempt to liberate α-synuclein prions from Lewy bodies, which are compact and densely packed intracellular structures, we partially purified prions from PD brain homogenates with 2% sarkosyl followed by limited proteolysis with proteinase K (PK) prior to PTA precipitation (PK/PTA).

**Fig. 1.**
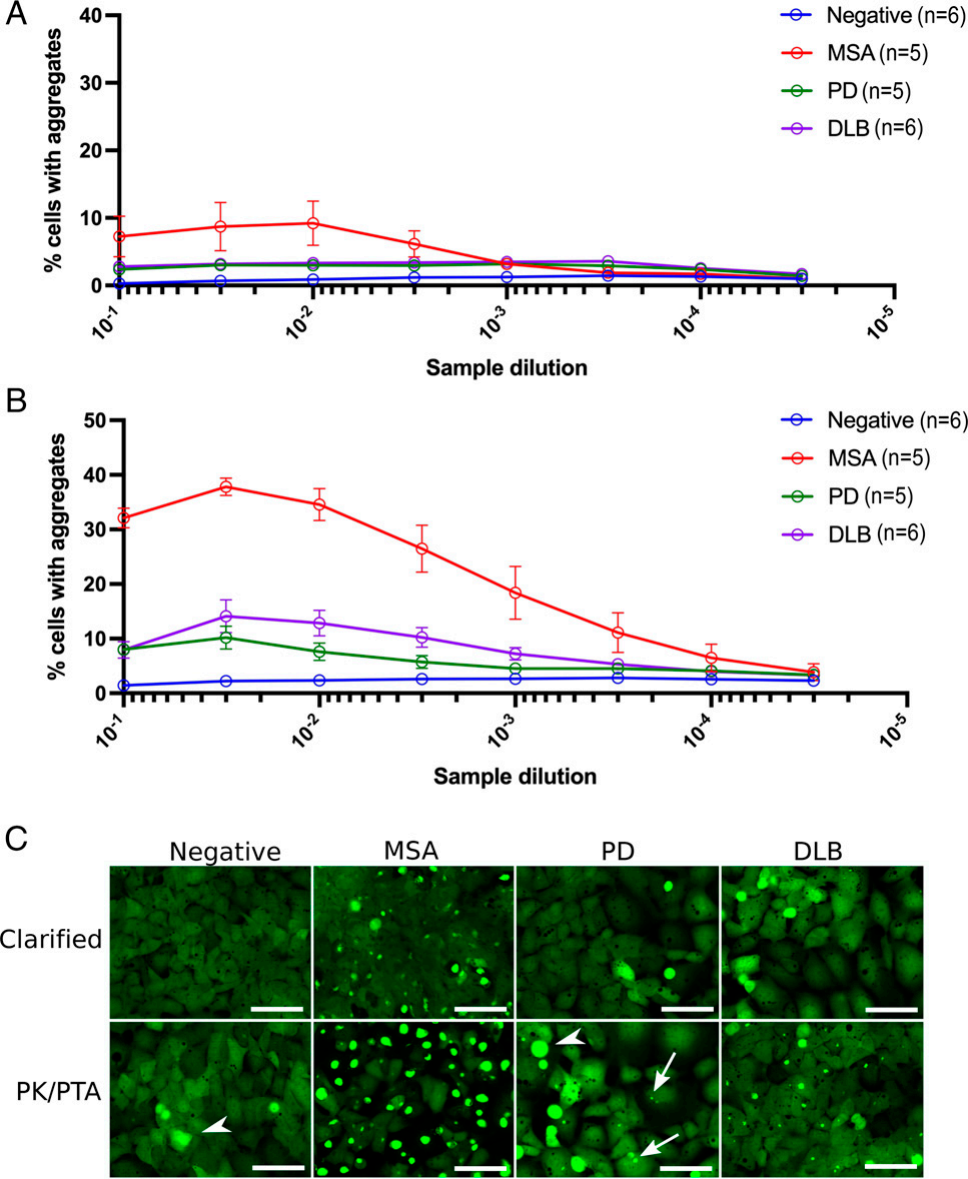
Effect of PK/PTA treatment on infectivity of human synucleinopathy samples in cultured HEK293T cells expressing syn140*A53T-YFP. (*A*) Cultured cells were infected with half-log dilutions of CBH from cognitively normal control brains (negative) and brains from MSA, PD, and DLB patients. Fluorescent puncta were measured 4 d following infection and presented as % cells with aggregates. The data points are the averages of signals from each disease cohort. (*B*) These same homogenates were treated with PK/PTA-precipitated samples and incubated in syn140*A53T-YFP cells to test for infectivity and measured 4 d following infection. The data revealed a significant increase in infectivity among the PK/PTA-precipitated fractions derived from MSA, PD, and DLB samples. The data points are the averages of signals from each disease cohort. (*C*) Representative images of syn140*A53T-YFP cells infected with the indicated preparations of negative control, MSA, PD, and DLB samples. The arrows point to small puncta measured in cells infected with PD inocula. Arrowheads indicate dead or dividing cells not counted as aggregates. YFP is shown in green (Scale bars: 100 μm).

To reliably measure α-synuclein prion infectivity from the brains of deceased patients with MSA, PD, or DLB, we performed a half-log dilution series of clarified brain homogenates (CBH) and of samples that had been PK/PTA precipitated. The CBH from MSA cases revealed a low level of infectivity at the highest concentrations of the sample (Fig. 1*A*). When homogenates from these same MSA samples were subjected to PK/PTA precipitation, 35% of the α-syn140*A53T-YFP cells formed puncta in the lowest dilutions. This contrasts with only 2.5% of cells treated with PK/PTA-precipitated samples from brain homogenates of cognitively normal human patients (referred to as negative controls) that developed puncta (Fig. 1*B*). Upon dilution, the PK/PTA-treated MSA samples displayed a robust dose–response relationship within this cell line. In addition to an increase in infectivity, the treatment also led to visibly larger puncta in the cells when compared to the inclusions induced by CBH (Fig. 1*C*).

### α-Synuclein Prion Infectivity in PD and DLB Brain Specimens.

Previously, PTA precipitation of PD samples was unsuccessful at uncovering prion activity when tested in α-syn140*A53T-YFP cells (16). We next decided to examine whether the PK/PTA precipitation, which greatly increased infectivity for MSA samples, would enable the measurement of prions derived from PD and DLB postmortem brains. We first tested these samples in α-syn140*A53T-YFP cells. With CBH from the amygdalae of five PD and six DLB patients, only ∼5% of cells developed puncta, and the measured response was unaffected by increasing dilutions (Fig. 1*A*). The low levels of α-synuclein prion activity in CBH from PD and DLB cases were consistent with our previously reported findings (16). The ability of these samples to induce formation of α-synuclein aggregates was markedly enhanced following PK/PTA precipitation (Fig. 1*B*). Although the increases in infectivity were modest when compared to MSA specimens, the DLB samples contained measurable levels of α-synuclein prions that were attenuated by dilution. When examined, the puncta in both the PD- and DLB-treated cells appeared as small punctate inclusions, though there were considerably fewer aggregates induced with PD (Fig. 1*C*). From these studies, we concluded that sarkosyl extraction followed by limited proteolysis and PTA precipitation substantially increased the α-synuclein prion infectivity from brain homogenates prepared from people who had died of α-synucleinopathies. Additionally, we found that partially purified α-synuclein prions from DLB propagate in HEK cells.

### α-Synuclein (E46K) Distinguishes Prion Strains.

To characterize α-synuclein prion activity from MSA brain samples, we infected several HEK cell lines overexpressing α-synuclein fused to YFP (18). While one cell line expressed WT α-synuclein, three others expressed human α-synuclein containing point mutations causing fPD. These three mutations are A30P, E46K, and A53T (18). As discussed in the Introduction, cells expressing E46K α-synuclein protein abolished formation of nascent MSA α-synuclein prions, while cells expressing WT, A30P, or A53T α-synuclein protein displayed a robust induction of fluorescent puncta upon infection with the same MSA samples (18). In contrast, recombinant α-synuclein fibrils were capable of infecting all cell lines tested. Based upon these results, we concluded that MSA α-synuclein prions are likely to be conformationally distinct from PD prions (18). However, due to the inability of homogenates from PD patients to infect these cell lines, we were unable to more conclusively test this hypothesis.

**Fig. 2.**
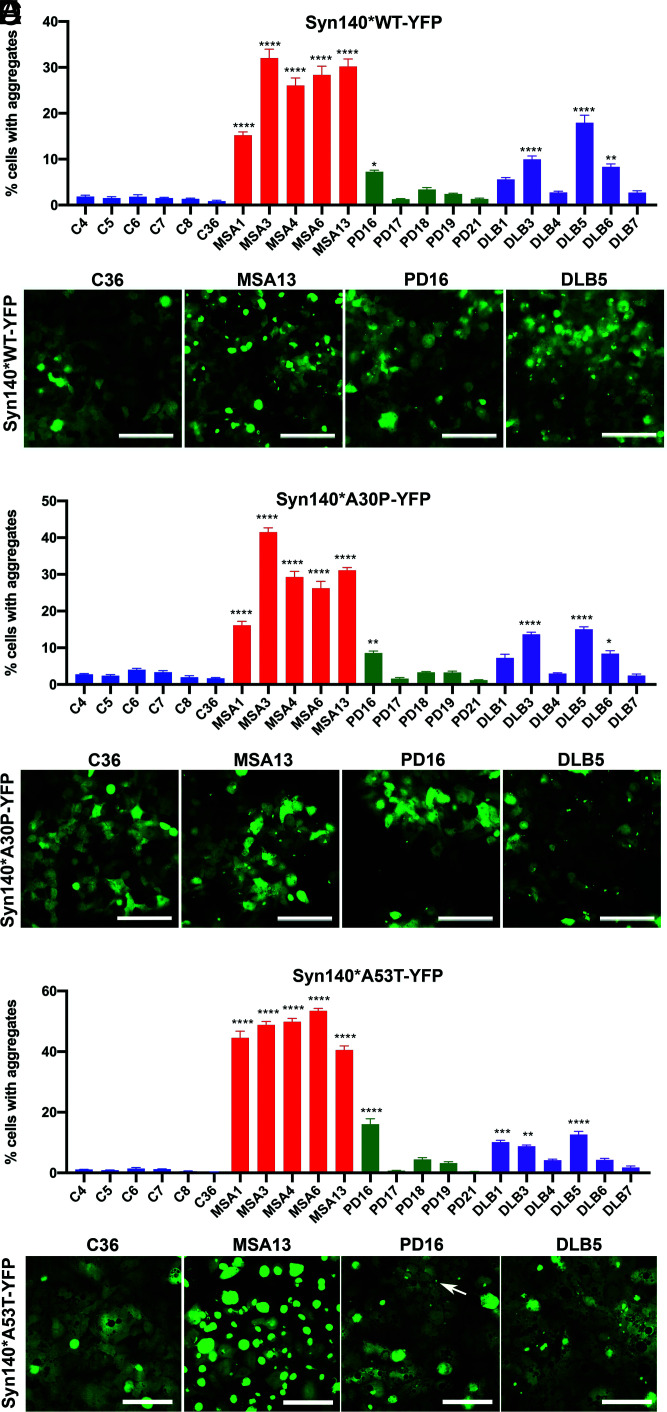
Effect of various synuclein substrates on cell infectivity of PK/PTA-precipitated preparations. (*A*) Syn140*WT-YFP cells reveal transmissibility of MSA, PD, and DLB α-synuclein prions. (*B*) Representative images of syn140*WT-YFP cells infected with PK/PTA-precipitated preparations of human samples. (*C*) Syn140*A30P-YFP cells were infected with PK/PTA-precipitated preparations from control, MSA, PD, and DLB cases. (*D*) Representative images displaying syn140*A30P-YFP puncta in infected cells. (*E*) Syn140*A53T-YFP cells reveal transmissibility of MSA, PD, and DLB α-synuclein prions. (*F*) Representative images of syn140*A53T-YFP cells infected with PK/PTA-precipitated preparations of human samples. Each sample was run in replicates of six wells. Data shown as mean ± SEM. Significance of each sample calculated against the mean of negative control samples. *P < 0.05, **P < 0.01, ***P < 0.001, ****P < 0.0001 (Scale bars: 100 μm).

Next, to ensure that PK/PTA precipitation did not alter the selectivity of MSA α-synuclein prions to these different HEK cell models and to characterize the putative activity of PD and DLB α-synuclein prions, we tested these samples in WT, A30P, and E46K cell lines. MSA PK/PTA samples (*n* = 5) retained their ability to infect HEK cells expressing α-syn140*WT-YFP, α-syn140*A30P-YFP, and α-syn140*A53T-YFP and displayed high levels of infectivity (Fig. 2 and Table 1). In comparison, infection of the same cell lines using PK/PTA samples from PD (*n* = 5) and DLB homogenates (*n* = 6) resulted in significant levels of infectivity from only one of the five PD samples (PD16), and in three of the six DLB samples (Fig. 2 and Table 1). Treatment with the PD/DLB samples resulted in lower levels of infectivity than treatment with MSA samples in all three cell lines. In addition, we also observed differences in the size of the aggregates induced in the three α-synuclein–expressing cell lines. The MSA samples were capable of inducing large punctate aggregates, whereas those induced by both the PD and DLB cases were noticeably smaller (Fig. 2 *B*, *D*, and *F*).

**Table 1.** Transmission of α-synuclein prions treated with limited proteolysis to cultured cells

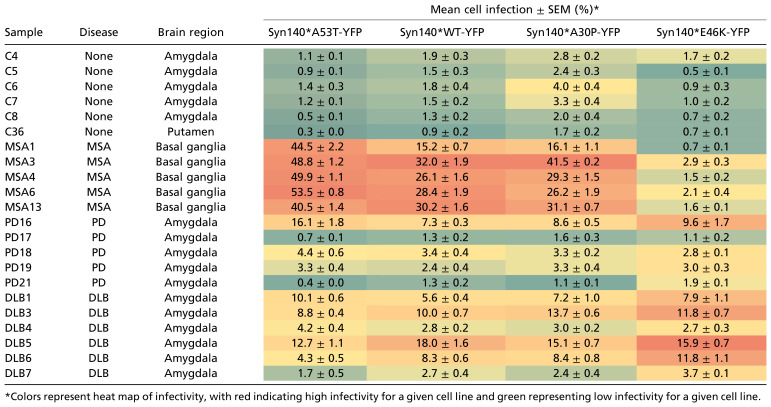

**Fig. 3.**
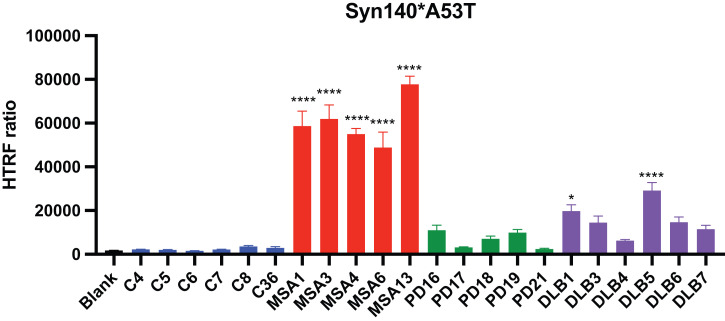
Cell infectivity of PK/PTA-precipitated preparations measured by HTRF in an untagged α-synuclein cell line. Infectivity of PK/PTA preps from samples were measured in α-syn140*A53T cells using an HTRF α-synuclein aggregation assay. Cells were incubated for 4 d with samples, lysed, and measured for α-synuclein aggregation. Data shown as mean ± SEM. Significance of each sample calculated against the mean of negative control samples. *P < 0.05, ****P < 0.0001.

Although we previously utilized cells expressing α-synuclein fused to YFP to measure α-synuclein prion levels (18), we remained concerned that the large YFP fluorophore might modify α-synuclein prion formation. The YFP fluorophore is composed of 235 amino acids and is fused to α-synuclein through an 18–amino-acid linker. Together, YFP and the linker are almost twice the size of α-synuclein. To address any potential artifact due to YFP and/or the linker, we generated an HEK293T stable cell line expressing the α-synuclein (A53T) protein lacking a fused YFP tag denoted α-syn140*A53T. To measure the induction of α-synuclein aggregation in this cell line, we used a homogenous time-resolved fluorescence (HTRF) assay that immunolabels α-synuclein prion aggregates. Upon infecting α-syn140*A53T cells with PK/PTA preps from control, MSA, PD, or DLB human brain samples, we measured α-synuclein prion aggregates using HTRF after 4 d of incubation (Fig. 3). Overall, the comparative levels of aggregation between samples were strikingly similar to those measured by YFP fluorescence in the α-syn140*A53T-YFP cells (Fig. 2*E* versus Fig. 3). This comparison demonstrates that the large YFP tag had little, if any, effect on the accumulation and detection of α-synuclein prions following HEK cell infections.

**Fig. 4.**
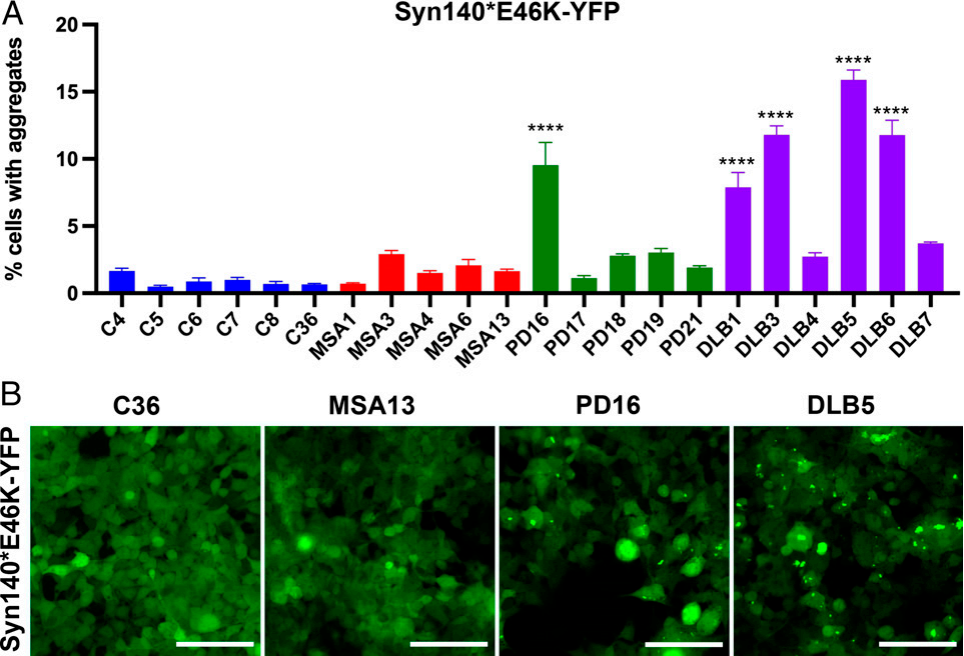
The E46K α-synuclein mutation allows replication of DLB prions. (*A*) Syn140*E46K-YFP cells reveal transmissibility of PD and DLB α-synuclein prions but show resistance to transmission of MSA α-synuclein prions. (*B*) Representative images of syn140*E46K-YFP cells infected with PK/PTA-precipitated preparations of human samples. Data shown as mean ± SEM. Significance of each sample calculated against the mean of negative control samples. ****P < 0.0001 (Scale bars: 100 μm).

As noted in the Introduction, we were previously unable to detect MSA prion infectivity in α-syn140*E46K-YFP cells (18). To test whether the PK/PTA procedure impacted this selectivity, we infected α-syn140*E46K-YFP cells with PK/PTA-treated MSA samples. Despite the robust increase in infectivity observed using other cell lines following PK/PTA precipitation, MSA α-synuclein prions were unable to infect cells expressing the E46K-mutated α-synuclein substrate (Fig. 4 and Table 1). However, when PK/PTA-precipitated α-synuclein prions from the PD and DLB samples were assessed in the α-syn140*E46K-YFP cells, we found that one of the five PD samples and four of the six DLB samples were capable of inducing significant aggregation when compared to controls. Furthermore, treatment with several of the DLB samples resulted in greater than 10% of the cells accumulating aggregates (Table 1).

**Fig. 5.**
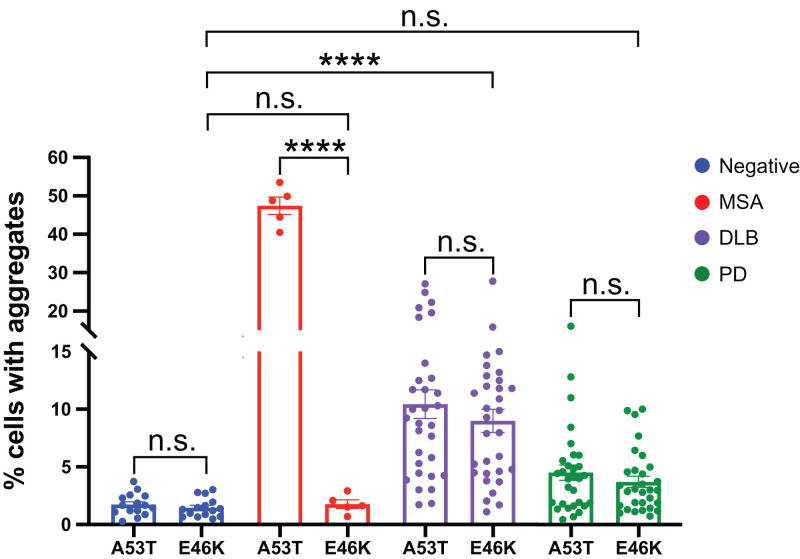
The E46K α-synuclein mutation differentiates MSA from DLB prions. An extended set of human patient samples was run through the syn140*A53T-YFP and syn140*E46K-YFP cell lines and plotted to visualize the effect of the α-synuclein substrate on infectivity. Data shown as mean ± SEM. Significance of each sample calculated against the mean of negative control samples. ****P < 0.0001. n.s., not significant.

To further characterize the infectivity of PD/DLB prions in α-syn140*E46K-YFP cells, we examined an expanded set of PD and DLB patient samples. A total of 30 individual PD samples and 31 individual DLB samples from the temporal cortex were subjected to PK/PTA precipitation followed by infection of both the A53T- and E46K-expressing cell lines. There was a significant decrease in the ability of MSA samples to infect cells expressing E46K compared to cells expressing A53T (*P* = 0.0001) (Fig. 5). In comparison, the increased set of DLB samples revealed no statistically significant difference in their ability to infect the two cell lines (*P* = 0.14) (Fig. 5). Although the low level of infectivity observed among our expanded set of PD samples prevented conclusive results, there was also no statistical difference in the infectivity observed in A53T- versus E46K-expressing cells (*P* = 0.68) (Fig. 5).

**Fig. 6.**
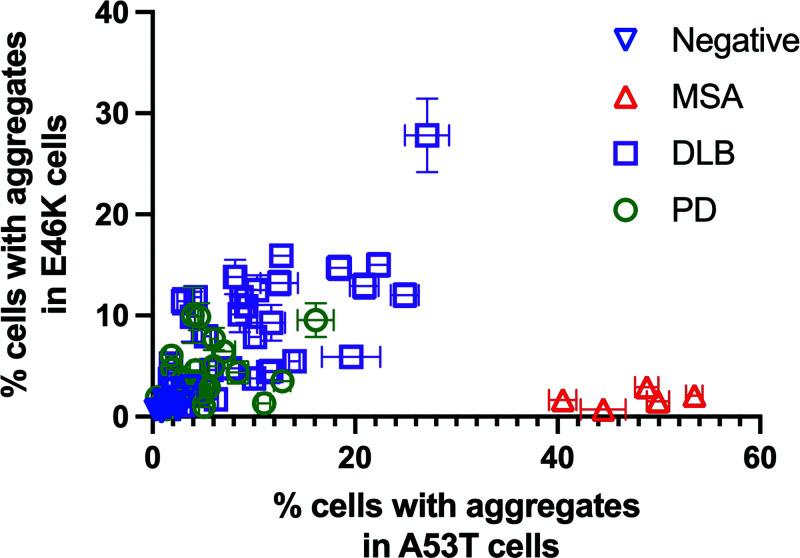
Permissiveness of the E46K versus A53T cell lines for each patient sample. Each patient sample is depicted in the graph by its ability to infect cells expressing the E46K mutation (*y* axis) in comparison to infectivity in cells expressing the A53T mutation (*x* axis).

To evaluate the α-synuclein prion infectivity in these two cell lines for a given sample, we also plotted the data from Fig. 5 in a scatter plot (Fig. 6 and *SI Appendix*, Fig. S1). By visualizing the data in this manner, it is obvious that most of the DLB and PD samples that resulted in infectivity in one cell line were also capable of infecting the other (Fig. 6 and *SI Appendix*, Fig. S1). MSA, however, is segregated from the DLB and PD samples as it is only able to infect the A53T-expressing cell line.

Based on earlier studies and the reported areas of high neuropathological α-synuclein burden in MSA, PD, and DLB cases (27), we created homogenates of amygdalae and temporal cortices from PD and DLB cases and basal ganglia from MSA cases for our experiments. To ensure that the inability of our MSA samples to infect α-syn140*E46K-YFP cells was not due to the tissue type used, we also tested homogenates of amygdalae from four of the five MSA postmortem patient brains. Although the amygdala is largely spared from pathological inclusions in MSA (27), we detected a robust induction of puncta (greater than 40% of cells) in α-syn140*A53T-YFP cells for two of the four samples (*SI Appendix*, Fig. S2). While infection in the WT and A30P α-synuclein expressing cells resulted in a decreased level of infectivity when compared to the A53T cells, these samples were unable to induce inclusions in the E46K α-synuclein–expressing cells (*SI Appendix*, Fig. S2). Our data indicate that the inhibitory effect of the E46K mutation on MSA infection is not dependent on the tissue type.

## Discussion

Transmission of α-synuclein prions from human brains to experimental hosts ranging from cultured cells to nonhuman primates was unsuccessful for decades (9). The first indication of transmissibility was a report that Lewy body pathology had been transmitted from the putamen of PD patients to fetal striatal tissue transplanted in the striatum (28–30). Since these fetal transplants proved ineffective as a treatment for advanced PD, few similar procedures have been subsequently performed. Notably, many experimental studies demonstrating α-synuclein prion infectivity have consisted of injecting recombinant α-synuclein fibrils, sometimes called preformed fibrils, into the brains of TgM83^+/−^ mice (31–33).

During our initial study of α-synuclein mutations, we found that MSA brain homogenates were infectious in both TgM83^+/−^ mice and HEK cells overexpressing α-synuclein containing the A53T mutation (15, 16, 19, 31). In parallel studies of homogenates from PD brains, we found little evidence for α-synuclein prion infectivity (16). Although PTA precipitation alone was sufficient for measuring MSA prion activity, it was unable to facilitate α-synuclein prion infectivity from PD brain homogenates (15, 16, 19). In other studies, detergent-insoluble fractions prepared from MSA and PD homogenates were added to HEK cells overexpressing α-syn*A53T fused to YFP; three of the five PD extracts and four of the five MSA extracts showed evidence for α-synuclein prion replication (21). Notably, the detergent-soluble fractions from MSA but not PD retained significant prion activity. Additionally, Recasens and colleagues isolated aggregates of α-synuclein from human PD brains using sucrose gradients, and upon intracerebral inoculation of these extracts into WT mice and macaque monkeys, a modest level of degeneration of nigrostriatal neurons was detected (34). Taken together, these studies raise the possibility that special conditions are needed to liberate PD α-synuclein prions sequestered inside Lewy bodies. Consistent with this hypothesis, ultrastructural characterization of Lewy bodies shows a crowded environment consisting of not only α-synuclein but also of membranes and organelles all densely packed into inclusions (35).

### Enhancing α-Synuclein Prion Infectivity.

We attempted to increase the α-synuclein prion infectivity in our samples by subjecting the brain homogenates to limited PK digestion followed by PTA precipitation. This protocol was initially developed to degrade proteins in brain homogenates and assist in the purification of PrP prions (36, 37). We hypothesized that this added step might enzymatically disrupt Lewy bodies and liberate α-synuclein prions, resulting in enhanced prion transmissibility. Surprisingly, digesting both MSA and DLB homogenates with PK followed by PTA precipitation resulted in increased infectivity in all MSA cases and a majority of the DLB cases.

Like the A53T point mutation in α-synuclein, the E46K mutation is also observed in fPD cases. When attempting to infect HEK cells expressing either of these mutations, we were surprised to find that unlike the A53T mutation, the E46K substitution inhibited de novo replication of MSA prions (18). To explore this unanticipated result, we asked whether the E46K point mutation, which prevented MSA prion propagation, might also inhibit DLB and/or PD prion replication. We were surprised when these studies showed that the E46K mutation did not inhibit either DLB or PD prion formation. While the low level of PD prion propagation in many samples made the results difficult to interpret, the much more robust replication of DLB prions is encouraging for future studies (Fig. 5).

### Structures of α-Synuclein Prions.

**Fig. 7.**
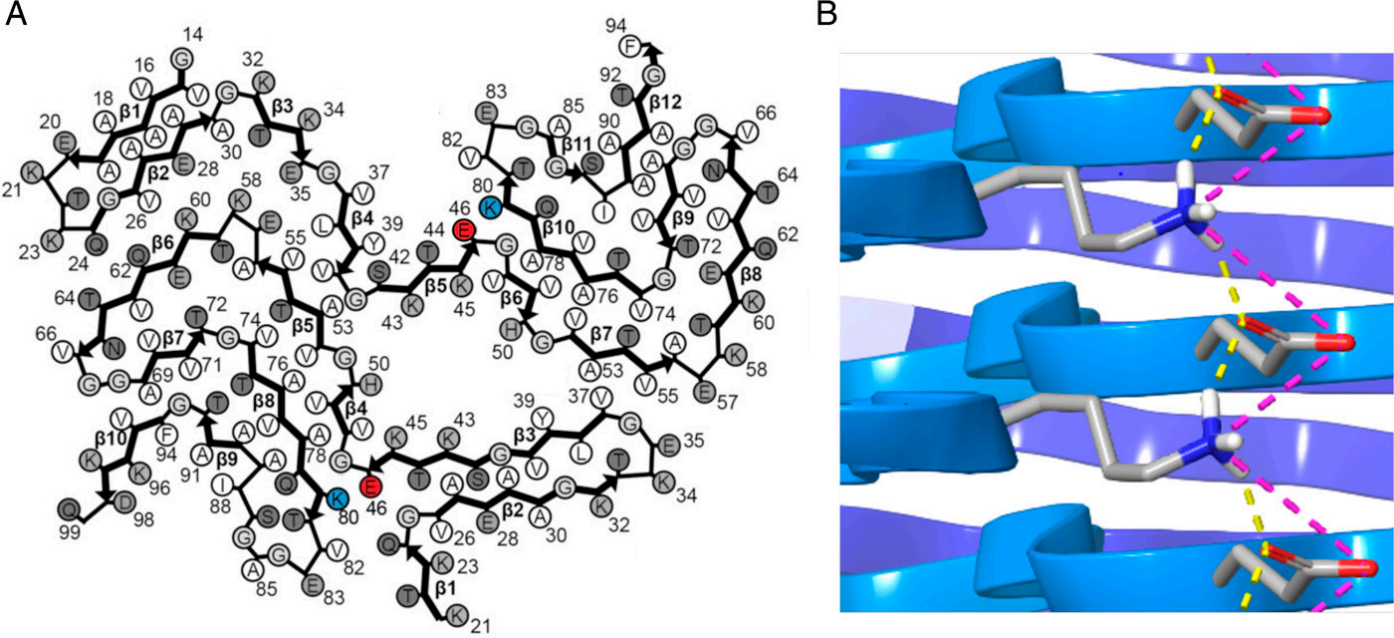
Interactions between E46 and K80 in MSA cryo-EM structures. (*A*) MSA type 1 α-synuclein filament (Protein Data Bank structure 6XYO) revealing close proximity of E46 (highlighted in red) and K80 (highlighted in blue) in both protofilaments. Adapted by permission from Springer Nature: ref. 38. (*B*) Proximity of E46 and K80 allow for the formation of hydrogen bonds (yellow lines) and salt bridges (pink lines).

The recent cryogenic electron microscopy (cryo-EM) structures of MSA α-synuclein filaments solved by Schweighauser and colleagues (38) provide insight into the molecular pathogenesis findings we report here. Two structurally different α-synuclein protofilaments were isolated from the brains of MSA patients. In both filaments, glutamic acid 46 is situated adjacent to the lysine at position 80 (Fig. 7). The close proximity of these two charged amino acid residues stabilizes the conformation of α-synuclein in MSA fibrils through a network of electrostatic and hydrogen-bonding interactions, both within a single protofilament and between protofilament layers. A mutation of the glutamic acid (E) residue at position 46 to a lysine (K) results in a loss of those favorable interactions, replacing them with an increase in localized positive charge that disfavors conformationally induced proximity between residue 46 and the lysine at position 80. This single residue appears sufficient to prevent the α-synuclein*E46K substrate from adopting any of the MSA protofibril conformations. Conversely, the ability of PD/DLB α-synuclein prions to induce aggregation in the α-syn140*E46K-YFP cells indicates that the glutamic acid at position 46 and the lysine at position 80 are not required to be in close proximity as new substrate is recruited into the infectious prion conformation.

PMCA and real-time quaking-induced conversion utilize sonication, shaking, and recombinant protein in a cell-free assay to study and measure α-synuclein aggregation from biological fluids and tissues (22, 23, 39, 40). Using these assays, distinct signatures of α-synuclein amyloid accumulation were observed using cerebrospinal fluid (CSF) from MSA and PD patients, leading the authors to conclude that these diseases are caused by distinct α-synuclein conformations (22). In contrast to our studies, which showed that α-synuclein prion activity of MSA was much greater than PD, PMCA of CSF from PD patients resulted in higher levels of aggregation when compared to MSA (22). The HEK cell assays used in studies by us and others, such as Marc Diamond’s laboratory, utilize physiological conditions (16, 18, 21). Furthermore, recent cryo-EM studies have revealed that fibrils composed of recombinant α-synuclein, tau, and amyloid-β are quite different from those solved from fibrils purified from primary patient samples (23, 41, 42). Though these two assays show tremendous value in studying synucleinopathies, the difference in their physiological relevance may produce disparate results.

Our studies and those of other investigators argue that PD/DLB prions are conformationally distinct from MSA prions. We measured a significant difference in the infectivity of PD and DLB samples in cells expressing A53T and E46K α-synuclein, with DLB samples appearing much more transmissible than those from PD. Though this could be attributed to a difference in the levels of α-synuclein prions that accumulate in these two diseases or the brain areas assayed, the possibility that PD and DLB patients accumulate distinct α-synuclein prion strains should be further explored. To address this, alternative assays using different α-synuclein mutants that support PD replication are required.

### Molecular Basis of PD, DLB, and MSA.

Distinguishing PD, DLB, and MSA has relied historically on clinical presentations and neuropathologic hallmarks, but that began to change with the discovery of point mutations in α-synuclein found to cause fPD (4). Another landmark advance occurred with the transmission of MSA α-synuclein prions to Tg(SNCA*A53T) mice (15). In contrast, neither prions from PD nor DLB brain homogenates could be transmitted to Tg mice or cultured cells expressing α-syn*A53T. Our discovery described here that the E46K α-synuclein point mutation is specifically permissive for DLB α-synuclein prion replication coupled with our earlier finding that E46K prevented MSA α-synuclein prion propagation (16, 18) offers diagnostic and possibly therapeutic opportunities.

Like Alzheimer’s disease, prevalence of the trio of α-synucleinopathies increases with age. At present, the α-synucleinopathies are thought to afflict more than 10 million people worldwide. Translating the foregoing advances into effective therapeutics remains an urgent goal.

## Materials and Methods

### Human Tissue Samples.

Frozen tissue samples were obtained from the Parkinson’s UK Brain Bank at Imperial College London, the neuropathology core of the Massachusetts Alzheimer’s Disease Research Center (ADRC), and the Banner Sun Health Research Institute’s Brain and Body Donation Program. Clinical reports were provided for most samples and summarized in Table 2.

**Table 2.** Demographic, clinical, and neuropathological characteristics of patient samples

Case	Country	Sex	Age at onset (y)	Duration (y)	Cause of death	Clinical diagnosis	Neuropathological diagnosis
C4							
C5							
C6							
C7							
C8							
C36							
MSA1	UK	M	78	8		Atypical akinetic-rigid syndrome with prominent ataxia; PSP questioned	MSA
MSA3	UK	F	52	6	Bronchopneumonia	MSA-P	MSA
MSA4	UK	M	68	7	Pneumonia	MSA	MSA
MSA6	UK	F	48	13	Pneumonia	Akinetic-rigid syndrome with antecollis and camptocormia: MSA versus PD	MSA
MSA13	USA	F	65	10	Chronic pneumonia	MSA	MSA
PD16	UK	M	70	6			PD
PD17	UK						PD
PD18	UK						PD
PD19	UK	M	78	9			PD
PD21	UK						PD
DLB1	UK	M	78	6			DLB
DLB3	UK	F	66	5			DLB
DLB4	UK						DLB
DLB5	UK						DLB
DLB6	UK						DLB
DLB7	UK						DLB

### Patient Neuropathology.

MSA and PD patient samples obtained from the Parkinson’s UK Brain Bank were bisected, with one hemisphere fixed in 10% (vol/vol) buffered formalin for diagnostic workup and the other coronally sliced, photographed on a grid, and then rapidly frozen. Blocks of tissue from 20 key anatomical areas were sampled from the fixed hemisphere. Sections from each area were stained with hematoxylin & eosin (H&E) and Luxol fast blue (LFB). For assessment and staging of neurodegenerative pathology, appropriate sections were stained with antibodies against α-synuclein, amyloid-β, tau, and p62. MSA was diagnosed based on the presence of oligodendroglial α-synuclein inclusions. PD cases were staged according to Braak criteria (43).

MSA patient samples obtained from the Massachusetts ADRC were bisected longitudinally. One-half was coronally sectioned and rapidly frozen, and the other one-half was fixed in 10% (vol/vol) neutral buffered formalin and then sectioned. Histological evaluation was performed on a set of blocked regions representative for a variety of NDs. All blocks were stained with LFB and H&E. On selected blocks, immunohistochemical analysis, including α-synuclein (mouse monoclonal antibody LB509; Life Technologies 18-0215), amyloid-β, and phosphorylated tau, was performed. The neuropathological diagnosis of MSA required the presence of GCIs (44).

Negative control, PD, and DLB tissues from the Banner Sun Health Research Institute were analyzed by complete gross and microscopic pathological examination using standard Arizona Study of Aging and Neurodegenerative Disorders methods and included pathologist assessment of both brain and peripheral organs (45).

### Tissue Homogenization and Preparation.

Brains were homogenized in calcium- and magnesium-free phosphate buffered saline (PBS) and then diluted to 10% (wt/vol). For CBH, samples were centrifuged at low speed (1,000 × *g*) to pellet cellular debris, and the supernatant was collected; these samples were designated CBH. For PK/PTA preps, crude brain homogenates were combined with sarkosyl to a final concentration of 2% (vol/vol) and benzonase to a final concentration of 150 U/mL and incubated at 37 °C for 2 h on a shaking incubator. PK was then added to the homogenates to a final concentration of 20 μg/mL and incubated for 1 h at 37 °C with agitation. To stop the PK reaction, phenylmethylsulfonyl fluoride was added to a final concentration of 1 mM. A 10% (wt/vol) solution of PTA at pH 7.0 was then added to a final concentration of 2% (vol/vol) to the sample and incubated overnight with shaking at 37 °C. Samples were then centrifuged for 30 min at 16,000 × *g*, and the pellet was resuspended in PBS.

### Cell Lines and Aggregation Assays.

Generation of HEK293T cell lines stably expressing α-syn140*WT-YFP, α-syn140*A53T-YFP, α-syn140*A30P-YFP, and α-syn140*E46K-YFP was performed as previously described (18). For generation of the untagged α-syn140*A53T–expressing HEK293T cell line used for HTRF, the same methods were used as described previously for generation of the other α-synuclein expressing cell lines (18). All α-synuclein expressing HEK293T cells were cultured and plated in 1× Dulbecco's modified Eagle medium supplemented with 10% (vol/vol) fetal bovine serum, 50 units/mL penicillin, and 50 μg/mL streptomycin (Thermo Fisher Scientific). Cells were maintained in a humidified atmosphere of 5% CO_2_ at 37 °C. Infectivity assays were performed by plating cell lines at cell densities between 3,500 and 4,500 cells per well in a 384-well plate with black polystyrene walls (Greiner) with 0.012 μg per well Hoechst 33342. Plates were returned to the incubator for 2 to 4 h. Samples were diluted in Dulbecco’s PBS (DPBS), mixed with an equal volume of Lipofectamine 2000 (Thermo Fisher Scientific) diluted in DPBS (3% vol/vol), and incubated for 1.5 h at room temperature. These samples were then added to the cells in six replicate wells (0.25% vol/vol final concentration of CBH and PK/PTA samples and 0.2% vol/vol final concentration of Lipofectamine 2000) and incubated for 3 d at 37 °C in a humidified atmosphere of 5% CO_2_. Individual plates were imaged using the IN Cell Analyzer 6000 cell-imaging system (GE Healthcare). DAPI and fluorescein isothiocyanate channels were used to collect two images from five different regions in each well. Raw images were analyzed with the IN Cell Developer software (GE Healthcare), using an algorithm designed to detect intracellular aggregates using pixel intensity and size thresholds in living cells, and quantified as % of cells with aggregates. To assess α-synuclein aggregation in the α-syn140*A53T–untagged cell line, the Alpha Synuclein Aggregation Kit was used as suggested by the manufacturer (Perkin-Elmer) and read on a PHERAstar FSX multimode plate reader (BMG LABTECH). The HTRF ratio was calculated by dividing the 665-nm signal by the 620-nm signal and multiplying by 10,000.

### Statistical Analysis.

Cell infection data are presented as mean ± SEM. Values represent averages of five images collected from each well of a 384-well plate. Technical replicates for each patient sample were averaged across six wells. Independent two-sample Student’s *t* tests were used to compare between groups (negative control, MSA, PD, and DLB) (Figs. 2–4). For data in Fig. 5, a linear mixed model of the outcome, % cells with aggregates, was used, utilizing a group by cell line interaction for the fixed effects via a cell-means model and random subject effect to account for up to six replicates per human patient sample. Kenward Rogers denominator degrees of freedom were used. Using estimate statements in SAS version 9.4, we compared within groups (negative control, MSA, PD, and DLB) across cell lines and compared each disease group to the negative controls for the E46K cell line. We assessed the plot of the residuals versus predicted and determined that transformation did not appear to improve model fit. Two-sided *P* values < 0.05 were considered statistically significant.

## Supplementary Material

Supplementary File

## Data Availability

All study data are included in the article and/or *SI Appendix*.
