# Supplementary material for: Different α-synuclein prion strains cause dementia with Lewy bodies and multiple system atrophy
Source: Proc Natl Acad Sci U S A. 2022 Feb 3;119(6):e2113489119. doi: 10.1073/pnas.2113489119 (PMC8833220; doi:10.1073/pnas.2113489119)
Supplement: Supplementary File [file pnas.2113489119.sapp.pdf]

PNAS
www.pnas.org

## Supplementary Information for

# Different α-Synuclein Prion Strains Cause Dementia with Lewy Bodies and Multiple System Atrophy

Jacob I. Ayers[1,2], Joanne Lee[1], Octovia Monteiro[1], Amanda L. Woerman[1,2], Ann A. Lazar[3], Carlo Condello[1,2], Nick A. Paras[1,2], and Stanley B. Prusiner[1,2,4*]

*To whom correspondence should be addressed: Stanley B. Prusiner, Institute for Neurodegenerative Diseases, University of California San Francisco, 675 Nelson Rising Lane, San Francisco, CA 94158; stanley.prusiner@ucsf.edu

**This PDF file includes:**

Figure S1
Figure S2

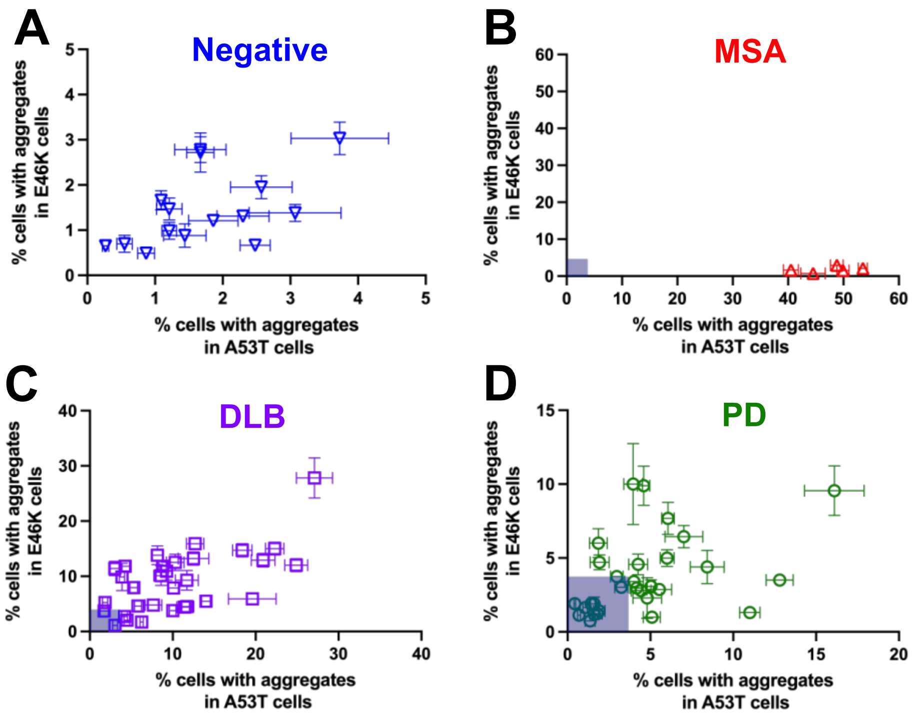


**Fig. S1.** To better visualize the data points from Figure 6, the **(A)** negative control, **(B)** MSA, **(C)** DLB, and **(D)** PD cohorts were each plotted in an individual graph. The blue boxes shown in panels B-D represent the variance observed among the negative control samples from panel A and the change in the range of axes among the different panels.

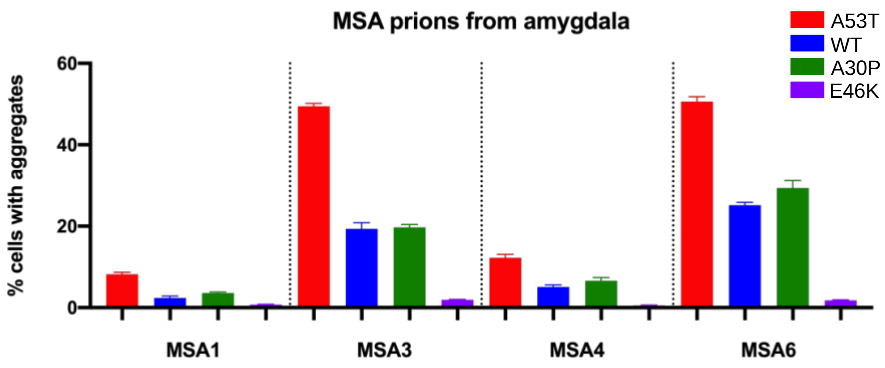


**Fig. S2.** Infectivity of MSA amygdala homogenates in α-synuclein expressing HEK cell lines. In contrast to previous data utilizing the basal ganglia from MSA cases, we also analyzed infectivity of MSA cases using amygdala sections. Amygdala samples from four of the five previously tested MSA cases were screened for infectivity in four α-synuclein expressing HEK cell lines. These samples behaved similarly to the MSA basal ganglia samples, in that they both revealed an inability to infect cells expressing E46K α-synuclein.
